# LncRNA MIR31HG targets HIF1A and P21 to facilitate head and neck cancer cell proliferation and tumorigenesis by promoting cell-cycle progression

**DOI:** 10.1186/s12943-018-0916-8

**Published:** 2018-11-20

**Authors:** Ru Wang, Zhihong Ma, Ling Feng, Yifan Yang, Chen Tan, Qian Shi, Meng Lian, Shizhi He, Hongzhi Ma, Jugao Fang

**Affiliations:** 1 Department of Otolaryngology Head and Neck Surgery, Beijing Tongren Hospital, Capital Medical University, Beijing, 100730 China; 2 Key Laboratory of Otolaryngology Head and Neck Surgery (Ministry of Education of China), Beijing Institute of Otolaryngology, Beijing, 100005 China; 3 Beijing Key Laboratory of Head and Neck Molecular Diagnostic Pathology, Beijing, 100730 China; 4 Department of Otolaryngology, Chengde Central Hospital, Chengde, 067000 Hebei China

**Keywords:** lncRNA MIR31HG, HIF1A, p21, Prognosis, Cell cycle, HNSCC

## Abstract

LncRNAs are involved in the initiation and progression of cancer. However, the molecular mechanism and diverse clinical prognosis of MIR31HG in head and neck squamous cell carcinoma (HNSCC) are still unclear. Our previous microarray analysis showed that lncRNA MIR31HG interacted with HIF1A may play an oncogenic role in laryngeal squamous cell cancer (LSCC). To determine whether lncRNA MIR31HG served as a poor prognosis factor and targeted HIF1A to facilitate cell proliferation and tumorigenesis in human HNSCC, we found MIR31HG and HIF1A were overexpressed in LSCC, MIR31HG overexpression or co-expression of HIF1A-positive and p21-negative could serve as a poor prognostic factor for LSCC patients. We further confirmed that MIR31HG promoted cell proliferation, cell cycle progression, and inhibited cell apoptosis in vitro and in vivo. The ingenuity pathway analysis and Western blot indicated that MIR31HG regulated cell cycle progression via HIF1A and p21 in HNSCC. The current results provide evidences for the role of MIR31HG in promoting HNSCC progression and identify MIR31HG as a prognostic biomarker and putative therapeutic target in HNSCC.

**Electronic supplementary material:**

The online version of this article (10.1186/s12943-018-0916-8) contains supplementary material, which is available to authorized users.

## Main text

Head and neck squamous cell carcinoma (HNSCC) is the sixth most common cancer worldwide, with about 650,000 new cases and 200,000 deaths annually [1]. Recently, there have been substantial improvements in multimodal approaches including surgery, chemotherapy, and radiotherapy. However, the 5-year overall survival (OS) rate has not increased significantly and the mortality rate has not decreased dramatically [2]. Thus, it is urgently needed to search for molecular biomarkers in the diagnosis and treatment of HNSCC.

Long non-coding RNAs (lncRNAs) are transcripts of length more than 200 nucleotides without protein-coding potential, regulating gene expression at the transcriptional, posttranscriptional, and epigenetic levels [3]. It is promising to investigate the functions and molecular mechanisms of dysregulated lncRNAs in HNSCC tumorigenesis and progression. MIR31HG (NCBI No: NR 027054) is a recently discovered long non-coding RNA, length of 2166 bp; its transcription is regulated by methylation of the promoter region [4]. Reportedly, MIR31HG plays oncogenic role and its overexpression can serve as a poor prognosis marker in several cancers, including oral cancer, breast cancer, and pancreatic ductal adenocarcinoma [5–7]. Accumulating study has revealed that MIR31HG can promote cancer initiation, progression, and metastasis by multiple mechanisms. MIR31HG inhibits the oncogene-induced cell senescence phenotype by regulating transcription of tumor suppressor p16 (INK4A) [8]. MIR31HG knockdown suppresses the capacity for proliferation, migration, and invasion of ESCC cells by targeting Furin and MMP1 [9]. Another study identified MIR31HG as a hypoxia-inducible lncRNA that formed a complex with HIF1A via direct binding and facilitating the recruitment of HIF1A and p300 cofactor for driving the progression of oral cancer [5]. However, the molecular mechanism, aggressive features, and diverse clinical prognosis of MIR31HG in HNSCC have not been fully understood. In our previous study, we screened lncRNA and mRNA expression profiles in LSCC tissues and found MIR31HG was positively correlated with HIF1A that plays an oncogenic role in LSCC [10]. To determine whether lncRNA MIR31HG served as a poor prognostic factor and targeted HIF1A to facilitate cell proliferation and tumorigenesis in human HNSCC, we analyzed the correlation between MIR31HG, HIF1A, and p21 expression and clinical prognosis. We conducted in vitro and vivo functional experiments and investigated the putative downstream pathway. The current results indicated that MIR31HG overexpression or co-expression of HIF1A-positive and p21-negative was correlated with the aggressive clinicopathological traits and served as a poor prognostic factor for LSCC patients. Moreover, MIR31HG facilitated cell proliferation and tumorigenesis via HIF1A and p21 by promoting the cell-cycle progression in HNSCC.

## Findings

### Overexpression of MIR31HG or co-expression of HIF1A-positive and p21-negative was correlated with aggressive clinicopathological traits and served as a poor prognostic factor for LSCC patients

**Fig. 1:**
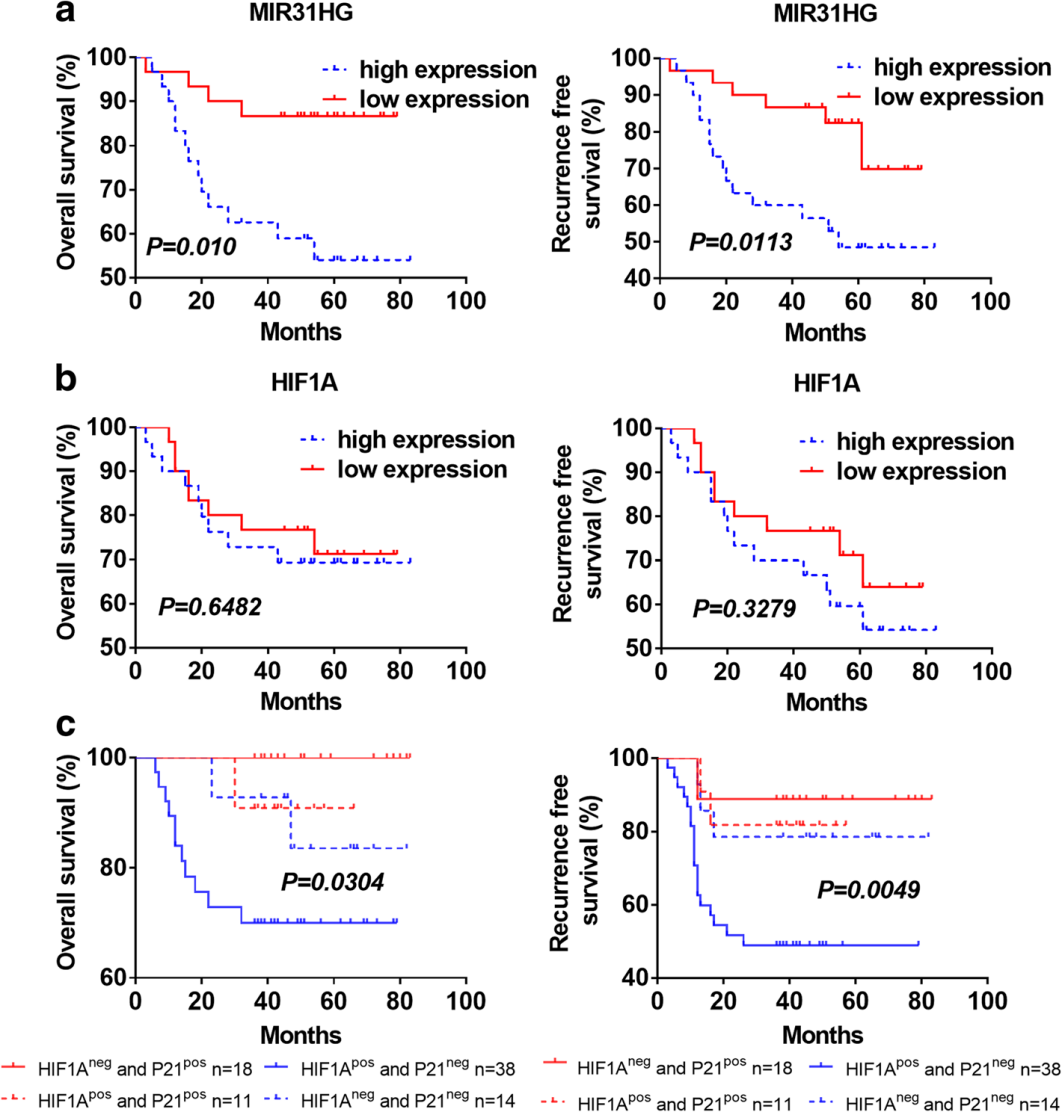
Overexpression of MIR31HG or co-expression of HIF1A-positive and p21-negative served as a poor prognostic factor for LSCC patients. **a** Patients in the MIR31HG high-expression group (*n* = 30) had a significantly worse overall survival and recurrence free survival than patients in the MIR31HG low-expression group (*n* = 30). *P* < 0.05, log-rank test. **b** Patients in the HIF1A high-expression group (*n* = 30) had a worse overall survival and recurrence free survival than patients in the HIF1A low-expression group (*n* = 30). *P* > 0.05, log-rank test. **c** Patients were stratified into four groups based on the expression of HIF1A and p21, patients with HIF1A-positive and p21-negative expression had a significantly worse overall survival and recurrence free survival than other groups. *P* < 0.05, log-rank test

We performed qRT-PCR to test the relative expression of MIR31HG and HIF1A in 60 pairs of LSCC cancer tissues and corresponding adjacent normal tissues. The results showed that MIR31HG and HIF1A were overexpressed in LSCC tissues (Additional file 1: Figure S1a, *P* < 0.05). qRT-PCR was also performed to test the expression of MIR31HG gene in plasma. It found that the expression of MIR31HG was higher in the early-stage and advanced LSCC plasma than that in the vocal polyp plasma (Additional file 1: Figure S1b, *P* < 0.05). To further investigate the relationship between MIR31HG expression level and clinicopathological traits, we divided the 60 patients into high- and low-MIR31HG expression groups according to the patients’ overall median MIR31HG expression level. The MIR31HG overexpression was significantly correlated with advanced T category and poor lymph node metastasis (Additional file 1: Table S1). Kaplan–Meier analysis showed significantly better OS and recurrence-free survival (RFS) rates in patients with lower MIR31HG expression than in patients with higher MIR31HG expression (Fig. 1a). However, no significant difference was observed between patients with lower and higher HIF1A expression (Fig. 1b). Additionally, the univariate and multivariate analysis identified high MIR31HG expression as an independent prognostic factor for LSCC patients (Additional file 1: Table S2). Thus, these data indicated that MIR31HG expression may exert a critical role in LSCC progression and metastasis and serve as a novel prognostic factor for LSCC patients. Compared with the study in oral cancer [5], our study, by contrast, used a larger tissue samples and plasma samples to validate that MIR31HG was a prognostic biomarker for HNSCC.

We further performed immunochemistry to test the level of HIF1A and p21 proteins in LSCC. The results showed that HIF1A was found primarily in the cytosol, whereas p21 was predominantly expressed in the nucleus (Additional file 1: Figure S2). The positive rate of HIF1A expression in LSCC was higher than that in vocal polyp. Oppositely, the positive rate of p21 in LSCC was lower (Additional file 1: Table S3–4, *P* < 0.05). Furthermore, the expression of HIF1A or p21 in LSCC was closely associated with the clinical T stage, lymph node metastasis, and tumor differentiation (P < 0.05). Spearman analysis indicated that HIF1A expression was inversely associated with that of p21 in LSCC tissues (*P* < 0.01, Additional file 1: Table S5). Kaplan–Meier analysis showed that patients with HIF1A-positive and p21-negative expression had poorer OS and RFS rate (*P* < 0.05, Fig. 1c). Moreover, multivariate Cox regression analysis showed that co-expression of HIF1A-positive and p21-negative was identified as a significant independent factor related to recurrence (Additional file 1: Table S6).

### MIR31HG promoted cell proliferation in vitro and vivo

**Fig. 2:**
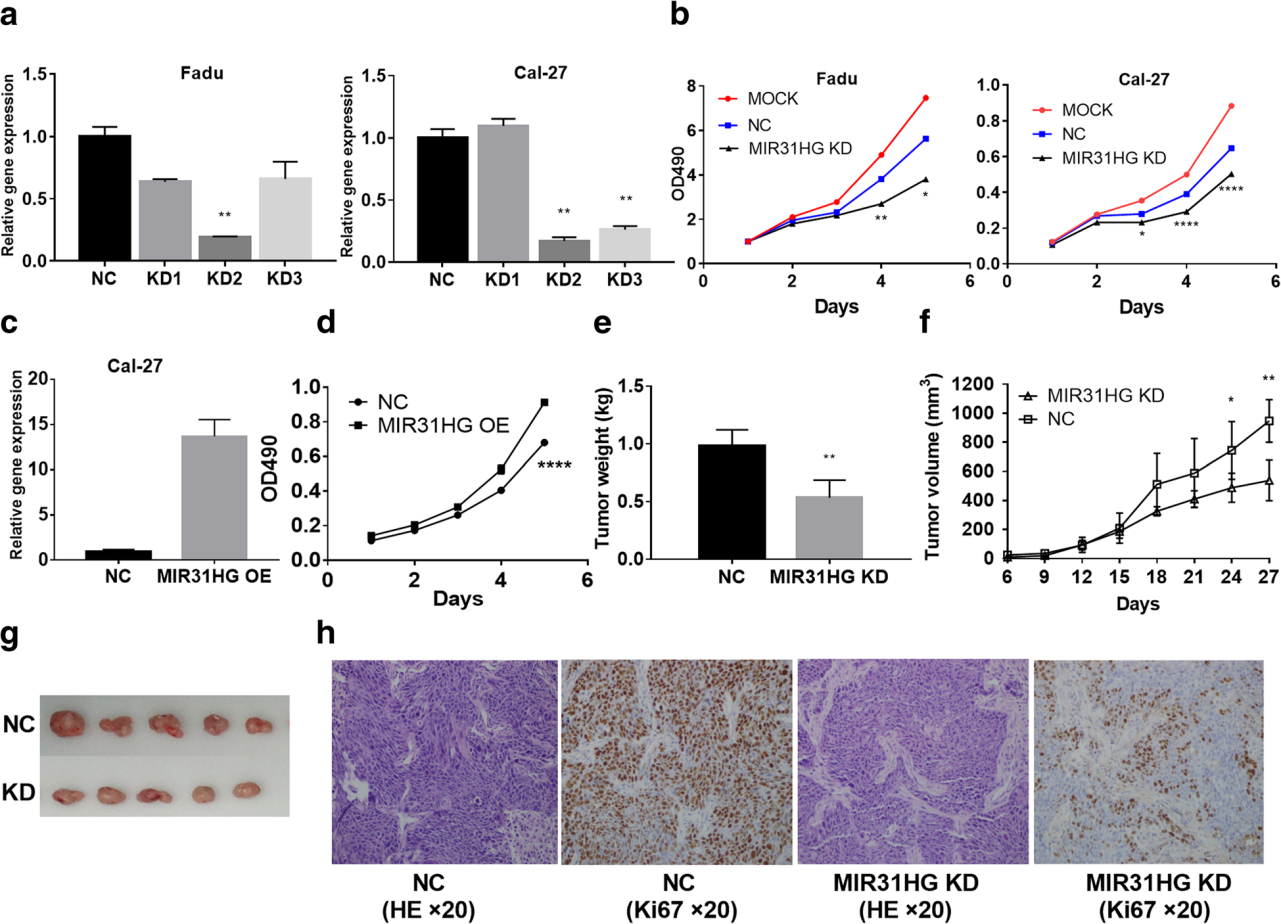
MIR31HG knockdown suppressed HNSCC cells proliferation in vitro and vivo. **a** qRT-PCR analysis of MIR31HG gene expression in FaDu and Cal-27 cells. **b** MTT assay of cell proliferation in MIR31HG knockdown or NC (transfected with negative control lentivirus) and MOCK (blank control) groups at indicated times. **c** qRT-PCR analysis of MIR31HG gene expression after MIR31HG overexpression (OE) lentivirus transfected in Cal-27 cells. **d** MTT assay of cell proliferation in MIR31HG OE or NC groups at indicated times. **e** The weights of the tumor in nude mice were calculated and compared. **f** The volumes of tumor in nude mice were calculated and compared. **g** Tumor images after all the mice were killed and tumors were removed. **h** Representative images of HE staining, immunohistochemical staining of Ki67. The results are presented as the mean ± SD for each group (*n* = 5). *P < 0.05, **P < 0.01, ****P < 0.0001 by Student’s t-test

We performed MTT assay and constructed HNSCC tumor xenograft model to evaluate the effect of MIR31HG on cell proliferation in vitro and vivo. First, we constructed three MIR31HG knockdown (KD) shRNAs to downregulate the expression of MIR31HG in FaDu and Cal-27 cells (Additional file 1: Figure S3). The KD2 shRNA decreased MIR31HG expression by 81% in FaDu cells and 83% in Cal-27 cells, indicating the high efficiency and stability of the transfection (Fig. 2a). Subsequently, the MTT assay displayed that MIR31HG KD significantly inhibited the growth of cancer cells (Fig. 2b). Then, we constructed MIR31HG overexpression (OE) lentivirus to upregulate the expression of MIR31HG in Cal-27 cells. The MIR31HG OE lentivirus increased MIR31HG expression by 13.667 times (Fig. 2c). The MTT assay displayed that MIR31HG OE promoted the growth of cancer cells (Fig. 2d). Moreover, we established a subcutaneously implanted tumor model in the nude mice by stably transfecting the FaDu cells with MIR31HG KD or NC lentivirus. After 4 weeks, the tumor volume in the MIR31HG KD group was remarkably smaller than that in the NC group and the tumor weight in the MIR31HG KD group was significantly lighter than that in the NC group (Fig. 2e-g). The Ki67 staining was decreased in xenografts of MIR31HG KD group compared with NC group. These results suggested that MIR31HG could promote HNSCC cells proliferation in vitro and vivo.

### MIR31HG targeted HIF1A and p21 to regulate cell cycle progression in HNSCC

**Fig. 3:**
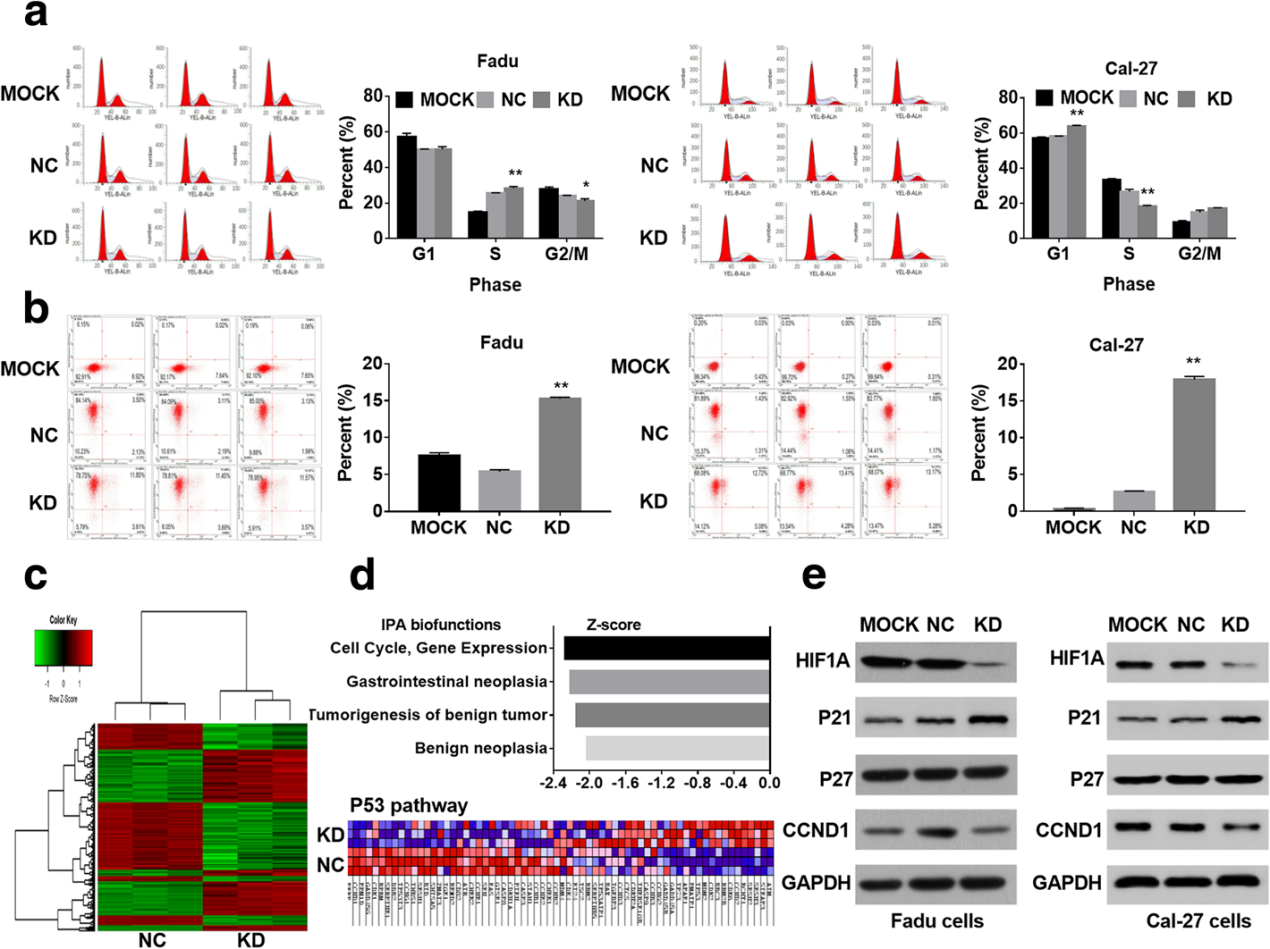
MIR31HG targeted HIF1A and p21 to regulate the cell cycle progression in HNSCC cells. **a** PI-FACS analysis of cell cycle distribution in FaDu and Cal-27 cells after MIR31HG shRNA transfection. Representative plots and cell percentage at different phases are illustrated. **b** Annexin V-APC-FACS analysis of cell apoptosis in FaDu and Cal-27 cells after MIR31HG shRNA transfection. Representative scatter plots and quantitative results are shown. **c** Differential mRNA expression profiling in MIR31HG KD and NC groups. Red color indicated overexpression and green color indicated low expression. Every column represented a tissue sample, and every row represented a mRNA probe. **d** Upper panel was the summary of ingenuity pathway analysis (IPA) biofunctions related to MIR31HG knockdown (fold-change ≥ 1.5, FDR < 0.05). Lower panel was p53 pathway heatmap indicated altered gene expression in p53 pathway after the MIR31HG knockdown. **e** Western blot of HIF1A and HIF1A-regulated targets in FaDu (left) and Cal-27 (right) cells treated with MIR31HG shRNA. *P < 0.05, **P < 0.01 by Student’s t-test

To elucidate the mechanism of MIR31HG underlying cell proliferation, we evaluated the cell cycle, cell apoptosis, and the differential gene expression in MIR31HG KD cells. PI-FACS analysis revealed that MIR31HG KD promoted cell cycle arrest at G1 or S phase (Fig. 3a). Interestingly, Annexin V-APC-FACS cell apoptosis analysis showed an increased number of apoptotic cells in the MIR31HG KD group (Fig. 3b). Furthermore, we found 345 differentially expressed mRNAs after MIR31HG shRNA transfected in FaDu cells, including 180 upregulated mRNAs and 165 downregulated mRNAs (Fig. 3c). The hierarchical clustering showed that differentially expressed mRNAs perfectly distinguished the MIR31HG KD cells from the NC cells. The ingenuity pathway analysis showed the differential expressed genes were significantly related to cell cycle (Fig. 3d). Western blot further validated that MIR31HG KD decreased the expression of HIF1A and CCND1 but increased expression of p21 (Fig. 3e).These results suggested that MIR31HG might target HIF1A and p21 to regulate the cell cycle progression and apoptosis in HNSCC (see materials and methods in Additional file 2). Different from the study in oral cancer [5], our study found another action mechanism of MIR31HG and HIF1A.

## Conclusions

In this study, for the first time, we showed that lncRNA MIR31HG was specifically overexpressed in HNSCC and found that the overexpression of MIR31HG or the co-expression of HIF1A-positive and p21-negative served as a poor prognostic factors for LSCC patients. LncRNA MIR31HG facilitated HNSCC cell proliferation and tumorigenesis via HIF1A and p21 by promoting cell-cycle progression and inhibiting cell apoptosis. Thus, the results implicated the key role of MIR31HG in HNSCC progression and identified MIR31HG as a prognostic predictor and putative therapeutic target in HNSCC.

## Additional files

Additional file 1: **Figure S1**. Relative gene expression of MIR31HG in LSCC cancer tissues and plasma. **Figure S2**. IHC of HIF1A and p21 proteins in LSCC tissues. **Figure S3**. MIR31HG KD lentivirus transfected Fadu and Cal-27 cells.** Table S1**. Association between MIR31HG and clinicopathological characteristics of LSCC patients. **Table S2** Multivariate analyses of factors associated with OS in LSCC patients. **Table S3**. Relationship of HIF1A expression and clinicopathological parameters in LSCC patients. **Table S4**. Relationship of p21 expression and clinicopathological parameters in LSCC patients.** Table S5**. The correlation of HIF1A with p21 expression in LSCC. **Table S6**. Multivariate analyses for recurrence free survival of the postoperative LSCC by COX regression. (RAR 8552 kb)

Additional file 2: Materials and Methods. (RAR 18 kb)

## Abbreviations

HNSCC: Head and neck squamous cell carcinoma
IHC: Immunohistochemistry
IPA: Ingenuity pathway analysis
KD: Knock down
LncRNAs: Long non-coding RNAs
LSCC: Laryngeal squamous cell carcinoma
NC: Negative control.
OS: Overall survival
RFS: Recurrence-free survival
